# Systematic review: brain metastases from colorectal cancer—Incidence and patient characteristics

**DOI:** 10.1186/s12885-016-2290-5

**Published:** 2016-04-01

**Authors:** Troels Dreier Christensen, Karen-Lise Garm Spindler, Jesper Andreas Palshof, Dorte Lisbet Nielsen

**Affiliations:** Department of Oncology, Herlev Hospital, University of Copenhagen, Herlev Ringvej 75, DK-2730 Herlev, Denmark; Department of Oncology, Aarhus University Hospital, Nørrebrogade 44, DK-8000 Aarhus C, Denmark

**Keywords:** Brain metastases, Colorectal cancer, Incidence, Lung metastases, *RAS* mutations

## Abstract

**Background:**

Brain metastases (BM) from colorectal cancer (CRC) are a rare event. However, the implications for affected patients are severe, and the incidence has been reported to be increasing. For clinicians, knowledge about the characteristics associated with BM is important and could lead to earlier diagnosis and improved survival.

**Method:**

In this paper, we describe the incidence as well as characteristics associated with BM based on a systematic review of the current literature, following the PRISMA guidelines.

**Results:**

We show that the incidence of BM in CRC patients ranges from 0.6 to 3.2 %. BM are a late stage phenomenon, and young age, rectal primary and lung metastases are associated with increased risk of developing BM. Molecular markers such as *KRAS*, *BRAF*, *NRAS* mutation as well as an increase in CEA and CA19.9 levels are suggested predictors of brain involvement. However, only *KRAS* mutations are reasonably well investigated and associated with an increased risk of BM.

**Conclusion:**

The incidence of BM from CRC is 0.6 to 3.2 % and did not seem to increase over time. Development of BM is associated with young age, lung metastases, rectal primary and KRAS mutation. Increased awareness of brain involvement in patients with these characteristics is necessary.

## Background

Worldwide, colorectal cancer (CRC) is the third most common cancer in men and second in women. CRC is the fourth most common reason for cancer-related death, and it is responsible for an estimated 8 % of deaths resulting from cancer [1].

Brain metastases (BM) are a common complication of lung cancer (40–50 % of cases), breast cancer (5–15 %), testicular cancer (10–15 %), and melanoma (10 %). BM from CRC are, however, relatively rare. BM are reported to develop late in the disease, and the patients normally have metastases to other organs before BM are diagnosed [2, 3]. The reported incidence of BM from CRC may be increasing because of improved diagnostics and increased survival of patients, but this is not well documented [2].

A diagnosis of BM is associated with increased morbidity and mortality. The reported median survival after a diagnosis of BM is 2.6 to 7.4 months, and only very few patients survive more than 1 year [4–6]. Intensified surveillance of patients at risk of BM development could potentially lead to earlier detection, hereby increasing the number of treatment options available and improving prognosis [4]. To identify patients at risk of developing BM, knowledge about patient characteristics associated with BM is important.

## Methods

We conducted a systematic review of the current literature, following PRISMA guidelines [5], to describe the incidence of BM from CRC, and to identify characteristics associated with increased risk of BM.

The complete search strategy in PubMed was ((brain AND (metastases OR metastasis)) OR (brain neoplasms AND (metastases OR metastasis)) OR cerebral metastasis OR cerebral metastases OR cerebellar metastasis OR cerebellar metastases OR CNS metastasis OR CNS metastases) AND (colorectal cancer OR colorectal neoplasms OR cancer of the colon OR cancer of the rectum OR adenocarcinoma colon OR adenocarcinoma rectum OR adenocarcinoma colorectal OR colonic carcinoma OR rectal carcinoma OR colonic neoplasm OR rectal neoplasm). In EMBASE, the search was conducted by combining subject headings brain metastases/with colorectal adenoma/or colorectal cancer/or colorectal carcinoma/or colorectal disease/or colorectal surgery/or colorectal tumor/or metastatic colorectal cancer.

No automatic filters were applied to the searches. We included pre-reviewed, human studies in English in patients with CRC in which the incidence of BM or characteristics of patients developing BM were reported. We excluded reviews, studies older than 1980, and studies comprising less than 25 patients with CRC. We also excluded studies with a mixed tumor population in which data from CRC patients were not presented separately. If two studies described the same cohort of patients, only the newest was included.

Full articles were obtained and analyzed when appropriate. Reference lists of retrieved relevant articles were screened for additional material. Two authors (TDC and DN) independently surveyed the literature. In case of ambiguity or disagreement, a verdict was reached by consensus.

In order to analyze incidence and patient characteristics, eligible studies were selected for pooling of data and calculation of weighted means. Studies were deemed eligible if they included all patients diagnosed with CRC and identified BM patients from this cohort. Studies were only eligible if a diagnosis of BM was made while the patients were alive. Studies were ineligible for pooling of data if they identified their BM patients from various populations consisting of selected patients with CRC, e.g. patients with metastatic CRC (mCRC), or if it was not clearly stated from what population patients with BM were identified. Weighted mean of incidence was calculated by dividing the sum of BM patients in relevant studies with the sum of CRC patients in the same studies. Weighted means of characteristics were calculated as the sum of BM patients with the specific characteristic in relevant studies divided by the sum of BM patients in those studies.

To compare stage of disease at primary diagnosis, Dukes and Astler-Coller classifications were translated to the TNM staging system (stage *A* = stage 1, stage *B* = stage 2, stage *C* = 3, and stage *D* = stage 4). If not stated in the studies, 95 % confidence intervals (95 % CI) were calculated for the incidence of BM, using the Clopper-Pearson method for binomial data. A 95 % CI was not possible to calculate for data with continuous outcome because most studies did not report the sampling variability.

## Results

**Fig. 1:**
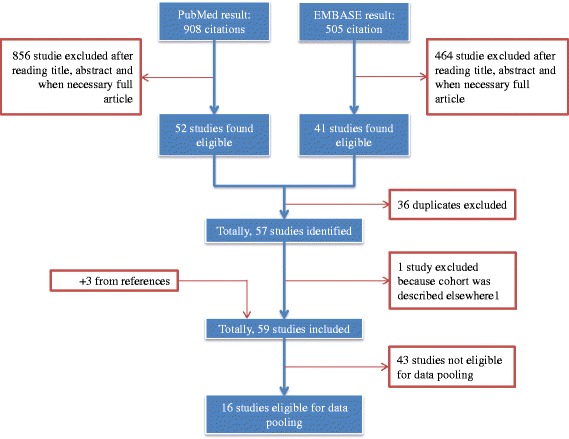
Consort diagram

The searches were conducted on April 15, 2015 (Fig. 1, consort diagram), and revealed 908 articles from PubMed and 505 from EMBASE. Totally, 93 studies were found eligible. Thirty-six were duplicates. Two studies described the same cohort of patients, and the oldest were excluded [6]. A further three relevant studies were identified from reference lists and included, increasing the total number to 59 studies (Table 1). All studies were retrospective. Thirty-one studies had consecutively included patients. The rest either did not include consecutively or did not clearly state how patients were included [4, 7–64].

**Table 1:** Studies included

Studies	Years	Country	No. CRC patients	Inci-dence	CI 95 %	Conse-cutive	Type of study^a^	Inclusion criteria for patients with CRC used in the studies.
Noura et al. [7]	1985–2006	Japan	2299	1.3 %	(0.8–1.8 %)	YES	Clinical	Diagnosed CRC
Suzuki et al. [8]	1979–2010	Japan	5345	2.6 %	(2.2–3.1 %)	-	Clinical	Diagnosed CRC
Hugen et al.^b^ [9]	1991–2010	Netherlands	5817	0.9 %	(0.7–1.2 %)	NO	Autopsy	Diagnosed CRC
Tan et al. [10]	1995–2003	Singapore	4378	0.6 %	(0.4–0.9 %)	YES	Clinical	Diagnosed CRC
Mongan et al. [11]	1984–2006	USA	1620	2.3 %	(1.7–3.3 %)	YES	Clinical	Diagnosed CRC
Jung M et al. [12]	1995–2008	Korea	8732	1.5 %	(1.2–1.7 %)	YES	Clinical	Diagnosed CRC
Pramateftakis et al. [13]	1990–2009	Greece	670	0.7 %	(0.2–1.7 %)	YES	Clinical	Diagnosed CRC
Jiang et al. [14]	1991–2010	China	8220	0.7 %	(0.6–0.9 %)	-	Clinical	Diagnosed CRC
Tevlin et al. [15]	1988–2012	Ireland	4219	0.3 %	(0.1–0.5 %)	-	Clinical	Diagnosed CRC
Tanriverdi et al. [16]	2001–2012	Turkey	4864	2.7 %	(2.3–3.2 %)	-	Clinical	Diagnosed CRC
Temple et al. [17]	1959–1979	USA	999	2.9 %	(2.0–4.1 %)	YES	Autopsy	Diagnosed CRC
Naito et al. [18]	1967–1992	Japan	778	1.9 %	(1.1–3.2 %)	YES	Clinical	Diagnosed CRC
Hammoud et al. [19]	1980–1994	USA	8632	1.7 %	(1.5–2.0 %)	YES	Clinical	Diagnosed CRC
Ko et al. [20]	1970–1996	Taiwan	7153	0.7 %	(0.6–1.0 %)	-	Clinical	Diagnosed CRC
Zorrilla et al. [21]	1996–2000	Spain	378	2.4 %	(1.1–4.5 %)	YES	Clinical	Diagnosed CRC
Schouten et al. [22]	1986–1998	Netherlands	720	1.4 %.	(0.7–2.5 %)	-	Clinical	Diagnosed CRC
Barnholtz-Sloan et al. [23]	1973–2001	USA	42,817	1.8 %	(1.7–2.0 %)	YES	Register	Diagnosed CRC
Kim et al. [24]	1987–2009	Korea	4350	1.1 %	(0.8–1.4 %)	-	Clinical	Diagnosed CRC^d^
Sundermeyer et al. [25]	1993–2002	USA	1020	3.2 %	(2.2–4.5 %)	-	Clinical	mCRC
Yeager et al. [26]	2008–2012	USA	918	4.0 %	(2.9–5.5 %)	-	Clinical	mCRC
Chyun et al. [27]	1977–1989	USA	78	23.0 %	(14.3–34.0 %)	YES	Clinical	mCRC
Patanaphan et al. [28]	1979–1982	USA	163	3.0 %	(0.7–6.2 %)	YES	Clinical	mCRC
Tran et al. [29]	1996–2009	Australia and USA	524	5.15 %	(3.4–7.4 %)	-	Clinical	mCRC
Hess et al. [30]	1994–1997	USA	984	0.7 %^c^	(0.3–1.5 %)	-	Clinical	mCRC
Tie et al. [31]	1999–2009	Australia	46	-		-	Clinical	mCRC
Khattak et al. [32]	2006–2011	Australia	2006	-^e^		NO	Clinical	mCRC^e^.
Kemeny et al. [33]	2003–2013	USA	169	5.0 %	(2.5–9.9 %)	NO	Clinical	Liver metastasectomy + chemotherapy
Yoshidome et al. [34]	1985–2001	Japan	207	4.0 %	(1.7–7.5 %)	YES	Clinical	Liver metastasectomy
de Jong et al. [35]	1982–2008	USA	1669	1.3 %	(0.8–2.0 %)	-	Clinical	Liver metastasectomy
Byrne et al. [36]	1987–2009	UK	1304	4.0 %	(3.0–5.2 %)	YES	Clinical	Liver metastasectomy
Higashiyama et al. [37]	1981–2001	Japan	100	13.0 %	(7.1–21.2 %)	YES	Clinical	Pulmonary metastasectomy
Hugen et al.^b^ [9]	1996–1999	Netherlands	1530	1.1 %	(0.6–1.8 %)	-	Clinical	Rectal cancer
Chiang et al. [38]	2002–2006	Taiwan	884	-^f^		YES	Clinical	Rectal cancer with T3 and T4 who had not received neoadjuvant therapy
Weiss et al. [39]	1944–1984	Multiple	1541	2.5 %	(1.8–3.4 %)	-	Autopsy	Colon cancer
Cascino et al. [40]	1977–1980	USA	1006	4.0 %	(2.9–5.4 %)	YES	Clinical	Colon cancer
Takagawa et al. [41]	1992–2003	Japan	638	1.3 %	(0.5–2.5 %)	YES	Clinical	Verified radical resected stages 1–3 tumor
van Gestel et al. [42]	2003–2008	Netherlands	5671	1.11 %	(0.9–1.4 %)	Yes	Register	Intended curatively treated primary cancer stages 1–3.
Tokoro et al. [43]	1998–2010	Japan	1364	1.8 %	(1.2–2.7 %)	YES	Clinical	Surgically treated for primary cancer and/or metastases
Scartozzi et al. [44]	1995–2005	Italy	99	-		-	Clinical	Both primary and metastases removed
Damiens et al. [4]	2000–2009	Canada	48	-		YES	Clinical	BM
Kruser et al. [45]	1994–2005	USA	49	-		-	Clinical	BM
Smedby et al. [46]	1987–2006	Sweden	1001	-		YES	Register	BM
Fokas et al. [47]	1996–2007	Germany	78	-		YES	Clinical	BM
Magni et al. [48]	2003–2013	Italy	41	-		Yes	Clinical	BM
Farnell et al. [49]	1976–1993	USA	150	-		YES	Clinical	BM
Kye et al. [50]	1997–2006	Korea	39	-		-	Clinical	BM and survived more than a month after BM diagnosis
Beak et al. [51]	2001–2009	Korea	118	-		-	Clinical	BM treated with WBRT, SRS, or surgery
Nieder et al. [52]	1983–2008	Norway	35	-		YES	Clinical	BM treated with WBRT
Bartelt et al. [53]	1985–2000	Germany	47	-		YES	Clinical	BM treated with WBRT
Heisterkamp et al. [54]	1989–2008	Germany	53	-		-	Clinical	BM treated with WBRT
Matsunaga et al. [55]	1992–2008	Japan	152	-		YES	Clinical	BM less than 3 cm and treated with SRS
Schoeggl et al. [56]	1993–1996	Austria	35	-		-	Clinical	BM treated with SRS
Wronski et al. [57]	1974–1993	USA	73	-		YES	Clinical	BM treated neurosurgically
Fowler et al. [58]	1999–2007	Australia	32	-		YES	Clinical	BM treated neurosurgically
Maglio et al. [59]	1999–2013	Italy	53	-		Yes	Clinical	BM treated neurosurgically
Mege et al. [60]	1998–2009	France	28	-		YES	Clinical	BM treated neurosurgically
Taher et al. [61]	1990–2009	Sweden	37	-		-	Clinical	BM treated neurosurgically
D’Andrea et al. [62]	1960–2000	Italy	44	-		-	Clinical	Single BM neurosurgically treated
Simonova et al. [63]	1992–1998	Czech republic	30	-		-	Clinical	Single BM radiosurgically treated
Onodera et al. [64]	1979–1998	Japan	1077	-		-	Clinical	Criteria unknown

*Abbreviations*: *CRC* Colorectal cancer, *mCRC* metastatic colorectal cancer, *BM* Brain metastases, *WRBT* Whole brain radiation therapy, *SRS* Stereotactic radiosurgery. Dash(−) means not reported

^a^Type of study: 1) Autopsy – studies where the patients were diagnosed based on autopsy 2) Clinic– studies where diagnosis are made radiological and authors had access to patient history, surgery reports and so on. 3) Register – studies with information from register and where authors did not have access to patient history or surgery reports

^b^The study by Hugen et al. contained information from two different cohorts. One cohort of patients with primary CRC in which diagnosis was based on autopsies, and a study based on radiological diagnosis that only included rectal cancer patients

^c^Hess et al. did only follow-up on patients once for the study, 4 months after referral to the hospital, which could result in lower incidence

^d^Kim et al. did not report characteristics of all 47 BM patients but only in 38 patients who received SRS treatment for BM

^e^Khattak et al. only report incidence of BM as only metastatic site of 0.4 %

^f^Chiang et al. did not report incidence from the entire cohort but only selected groups, e.g. patients with lung metastases

Forty-one studies either identified BM patients from patient populations that did not include all patients diagnosed with CRC, or did not report from what population patients with BM were identified, and were not eligible for pooling of data. Eighteen studies identified patients with BM from populations including all patients diagnosed with CRC (Table 1). Two of the 18 studies were autopsy studies and were therefore excluded from further analysis, giving a total of 16 studies eligible for data pooling [7, 8, 10–16, 18–24]. In all 16 studies, follow-up on CRC patients continued until death or end of study period, and none performed routine follow-up screening for BM.

### Incidence of BM in patients diagnosed with CRC

**Fig. 2:**
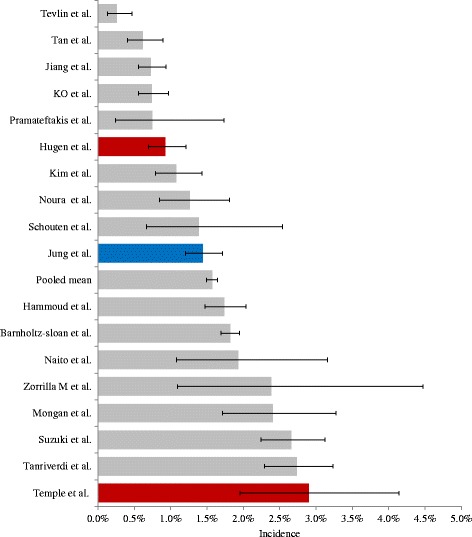
Incidence of brain metastases in patients with colorectal cancer. Incidence of brain metastases (BM) from colorectal cancer (CRC) in the 19 studies that identified patient with BM from populations including all patients diagnosed with CRC. Error bars indicate 95 % confidence interval. Gray: Studies with radiologically diagnosed brain metastases (17). Red: Autopsy studies (2). Blue: Pooled mean based on studies with radiologically diagnosed brain metastases

All 18 studies (Table 1 and Fig. 2) with patients diagnosed with CRC reported an incidence of BM between 0.6 and 2.9 % [7–24]. In the 16 studies eligible for pooling of data, the total number of CRC patients was 100,825 and the number of BM patients was 1588, resulting in an incidence of 1.55 % (95 % CI 1.48–1.63 %).

**Fig. 3:**
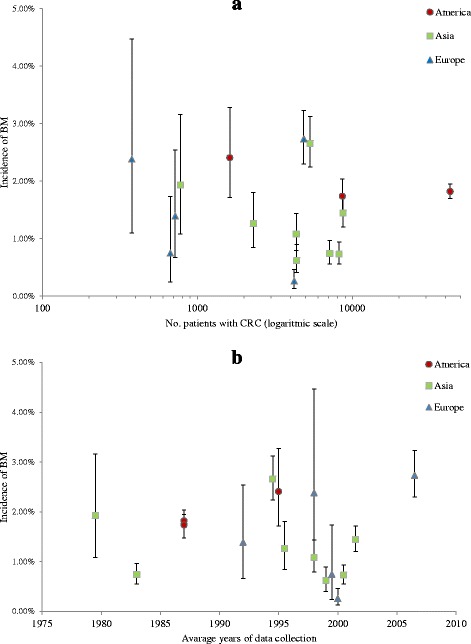
Incidence according to no. patients, years and region of data collection. Incidence of brain metastases (BM) in the 17 studies that included all patients diagnosed with colorectal cancer (CRC). Error bars indicate 95 % confidence interval. **a** - Incidence of BM according to size of cohorts. Studies sorted by regions. **b** - Incidence of BM from CRC according to average year of data collection. Studies sorted by region

The variation in the reported incidence between the 16 studies seemed to depend on sample size, with the highest variation among studies with the fewest patients (Fig. 3a). The reported incidence was also affected by region, with Asian studies reporting a lower incidence (weighted mean = 1.21 %) than the American (weighted mean = 1.82 %) and European (weighted mean = 1.55 %). The variation in incidence did not seem to be explained by different years of data collection (Fig. 3b).

Two autopsy studies reported the incidence of patients diagnosed with CRC. The incidence was 2.7 % in an American study which included all patients diagnosed with CRC at a single hospital between 1959 and 1979 [17]. In a more recent Dutch autopsy study, the incidence was 0.93 %. However, brain autopsies were not performed on all patients, which probably led to BM being underestimated [9].

### Incidence of BM in other CRC populations

Several studies reported an incidence from various CRC populations (Table 1), including three that also reported an incidence in all patients with CRC [9, 13, 24].

Nine studies reported an incidence of BM in a cohort of metastatic CRC (mCRC) patients. However, Hess et al. collected data, at a single time point, 4 months after referral of the patient to their hospital, and found an incidence of 0.71 %, which probably reflected the short follow-up [30]. Excluding this study, the incidence in patients with mCRC was 2.5 to 23 % [9, 12, 23, 25–29].

Four studies reported on a cohort of patients that had undergone liver metastasectomy, with incidences between 1.3 and 5 % [33–36], and a single study reported an incidence of 13 % after removal of pulmonary metastases [37].

Two studies included patients with rectal cancer only. Hugen et al. reported an incidence of 1.1 % in a cohort of 1530 patients with rectal cancer. However, the study only included metastatic sites at first diagnosis of metastatic disease, probably causing an underestimation of incidence [9]. Chiang et al. included patients with radically resected T3 or T4 rectal cancer. They reported an incidence of BM after lung metastases of 22.6 %, of 3.6 % after liver metastases, and of 2.9 % after local metastases [38].

Two studies included patients with colon cancer only. The incidence was 2.5 % in an autopsy study of patients who were diagnosed with colon cancer and had a necropsy performed at one of 16 hospitals [39]. The other study included all colon cancer patients treated at one hospital and found an incidence of 4 % [40].

Two studies reported an incidence of 1.1 to 1.3 % in patients who had been surgically treated for stage 1–3 primary cancer [41, 42]. And the last study reported an incidence of 1.8 % in patients who previously had surgically removed primary tumor or metastases [43].

### Characteristics of patients with brain metastases

The majority of the 59 studies reported clinicopathological characteristics of BM patients (Table 2), but only a few analyzed a statistical association. Of the 16 studies, eligible for pooling of data, only 14 described the characteristics of all included BM patients [7, 8, 10–16, 18–21, 23].

**Table 2:** Characteristics of patients with brain metastases in all studies

Article	Years	No. CRC patients	No. BM patients	Median age (years)	Median BMFI (month)	Male	Rectal primary	Extracranial metastases	Lung metastases	Liver metastases
Characteristics of patients with brain metastases in studies including all patients diagnosed with colorectal cancer.										
ᅟNoura et al. [7]	1985–2006	2299	29	61.1 (mean)	34.3	79 %	59 %	79 %	69 %	24 %
ᅟSuzuki et al. [8]	1979–2010	5345	142	61	22.8	61 %	43 %	78 %	66 %	42 %
ᅟHugen et al. [9]	1991–2010	5817	54	-	-	-	41 %	-	-	-
ᅟTan et al. [10]	1995–2003	4378	27	66	27.5	52 %	56 %	93 %	82 %	51.9 %
ᅟMongan et al. [11]	1984–2006	1620	39	-	25	54 %	43 %	100 %	78 %	
ᅟJung et al. [12]	1995–2008	8732	126	-	28.7	62 %	63 %	92.3 %	72 %	32.5 %
ᅟPramateftakis et al. [13]	1990–2009	670	5	55.7	7.5 (mean)	80 %	20 %	-	-	80 %
ᅟJiang et al. [14]	1991–2010	8220	60	62.5	21	60 %	50 %	88 %	65 %	30 %
ᅟTevlin et al. [15]	1988–2012	4219	11	73	24	73 %	27 %	73 %	-	-
ᅟTanriverdi et al. [16]	2001–2012	4864	133	-	31	53 %	56 %	89 %	65 %	51 %
ᅟTemple et al. [17]	1959–1979	999	29	-	-	62 %	41 %	-	86 %	76 %
ᅟNaito et al. [18]	1967–1992	778	15	-	22	53 %	67 %	-	80 %	-
ᅟHammoud et al. [19]	1980–1994	8632	150	61	26	62 %	41 %	95 %	71 %	52 %
ᅟKo et al. [20]	1970–1996	7153	53	-	33	74 %	62 %	77 %	54 %	26 %
ᅟZorrilla et al. [21]	1996–2000	378	53	61	30	44 %	55 %	100 %	67 %	67 %
ᅟSchouten et al. [22]	1986–1998	720	9	-	-	-	-	-	-	-
ᅟBarnholtz-Sloan et al. [23]	1973–2001	42,817	779	-	-	53 %	-	-	-	-
Characteristics of patients with brain metastases in studies including various selected cohorts of colorectal cancer patients.										
ᅟKim et al. [24]	1987–2009	4350	38^a^	-	-	66 %	58 %	5 %	-	-
ᅟSundermeyer et al. [25]	1993–2002	1020	33	-	-	-	-	-	-	-
ᅟYeager et al. [26]	2008–2012	918	37	-	-	-	-	-	-	-
ᅟChyun et al. [27]	1977–1989	78	18	-	28	50 %	-	95 %	55 %	22 %
ᅟPatanaphan et al. [28]	1979–1982	163	4	-	33	-	-	-	-	-
ᅟTran et al. [29]	1996–2009	524	27	-	-	-	-	-	-	-
ᅟHess et al. [30]	1994–1997	984	7	-	-	-	-	-	-	-
ᅟTie et al. [31]	1999–2009	46	46	-	-	-	-	-	-	-
ᅟKhattak et al. [32]	2006–2011	2006	10	-	-	-	-	-	-	-
ᅟKemeny et al. [33]	2003–2013	169	9	-	-	-	-	-	-	-
ᅟYoshidome et al. [34]	1985–2001	207	8	-	-	-	-	-	75 %	-
ᅟde Jong et al. [35]	1982–2008	1669	22	-	-	-	-	-	-	-
ᅟByrne et al. [36]	1987–2009	1304	52	-	40	56 %	-	90 %	81 %	29 %
ᅟHigashiyama et al. [37]	1981–2001	100	13	-	-	-	-	-	-	-
ᅟHugen et al. second cohort [9]	1996–1999	1530	17	-	-	-	-	-	-	-
ᅟChiang et al. [38]	2002–2006	884		-	-	-	-	-	-	-
ᅟWeiss et al. [39]	1944–1984	1541	38	-	-	-	-	-	68 %	74 %
ᅟCascino et al. [40]	1977–1980	1006	40	60	25	60 %	-	98 %	85 %	50 %
ᅟTakagawa et al. [41]	1992–2003	638	8	-	-	-	-	-	-	-
ᅟvan Gestel et al. [42]	2003–2008	5671	63	-	-	-	-	78 %	-	-
ᅟTokoro et al. [43]	1998–2010	1364	25	-	25	52 %	48 %	92 %	92 %	68 %
ᅟScartozzi et al. [44]	1995–2005	99	5	-	-	-	-	-	-	-
ᅟKruser et al. [45]	1994–2005	49	49	66	23	67 %	14 %	82 %	47 %	33 %
ᅟSmedby et al. [46]	1987–2006	1001	1001	-	25	-	-	-	-	-
ᅟFokas et al. [47]	1996–2007	78	78	-	20	39 %	-	64 %	-	-
ᅟMagni et al. [48]	2003–2013	41	41	58	36	61 %	59 %	95 %	88 %	37 %
ᅟFarnell et al. [49]	1976–1993	150	150	65	23	58 %	30 %	81 %	57 %	29 %
ᅟDamiens et al. [4]	2000–2009	48	48	63	24	52 %	48 %	90 %	64 %	50 %
ᅟKye et al. [50]	1997–2006	39	39	59	32 (mean)	59 %	56 %	97 %	80 %	41 %
ᅟBeak et al. [51]	2001–2009	118	118	58	-	53 %	61 %	-	75 %	45 %
ᅟNieder et al. [52]	1983–2008	35	35	-	-	-	-	-	-	-
ᅟBartelt et al. [53]	1985–2000	47	47	-	23	-	51 %	100 %	50 %	-
ᅟHeisterkamp et al. [54]	1989–2008	53	53	-	-	47 %	36 %	77 %	-	-
ᅟMatsunaga et al. [55]	1992–2008	152	152	-	27	67 %	42 %	74 %	61 %	33 %
ᅟSchoeggl et al. [56]	1993–1996	35	35	-	28	66 %	-	63 %	57 %	46 %
ᅟWronski et al. [57]	1974–1993	73	73	61	28	41 %	40 %	-	74 %	49 %
ᅟFowler et al. [58]	1999–2007	32	32	66	28	66 %	31 %	-	41 %	44 %
ᅟMaglio et al. [59]	1999–2013	53	53	65	-	42 %	43 %	96 %	75 %	-
ᅟMege et al. [60]	1998–2009	28	28	62	-	46 %	46 %	-	36 %	29 %
ᅟTaher et al. [61]	1990–2009	37	37	63	35	54 %	-	-	-	-
ᅟD’Andrea et al. [62]	1960–2000	44	44	53 (mean)	26	75 %	-	-	-	-
ᅟSimonova et al. [63]	1992–1998	30	30	-	-	-	-	-	-	-
ᅟOnodera et al. [64]	1979–1998	1077	17	-	-	77 %	71 %	88 %	76 %	47 %

*Abbreviations*: *CRC* Colorectal cancer, *BM* Brain metastases, *BMFI* brain metastases free interval (interval from primary diagnosis to BM development). Dash(−) means not reported

^a^Kim et al. did not report characteristics of all 47 BM patients but only in 38 patients who received SRS treatment for BM

### Timing of brain metastases

The median age at BM diagnosis ranged from 56 to 73 years. Four studies reported a median age higher than 65 years, and four studies reported it to be less than 60 years. In seven studies eligible for pooling of data, the age ranged between 55.7 and 73 years, and only two reported a median age higher than 65 years. Only seven studies reported that the age at primary CRC diagnosis in patients with BM ranged from 54 to 70 years [10, 15, 18, 27, 51, 57, 58].

The interval from primary CRC diagnosis to BM diagnosis (BM-free interval = BMFI) was between 20 and 40 months in a total of 28 studies, and between 21 months and 34.3 months in 11 studies eligible for pooling of data.

The BMFI after diagnosis of mCRC was 9–23 months [12, 21, 51], 5–12 months after lung metastases [11, 38, 57], and 7.4–25 months after liver metastases [10, 36, 38]. There was no significant association between primary tumor location and BMFI, but a tendency was noted toward a shorter interval in patients with rectal tumor [53]. BMFI was statistically associated with the treatment received between primary diagnosis and BM [19].

Barnholtz-Sloan et al. showed in their cohort of 42,817 CRC patients that the incidence proportion was statistically significantly higher in patients aged 50–59 (2.8 %), compared to patients aged 40–49 (2.4 %) and 60–69 (2.2 %) [23]. Nieder et al. reported an increase in BMFI in patients from the 1980s (6.5 months) to 2000s (31 months) [52].

### Gender

Thirty-seven studies reported the gender of BM patients. In these studies, 39 to 80 % were male. In 14 studies eligible for pooling of data, between 44 and 80 % of BM patients were male and the weighted mean was 57.2 %.

The only study that examined the association between gender and BM was Barnholtz-Sloan et al., who reported a borderline significant higher incidence in male patients (1.9 %) than in female patients (1.7 %), but their study design did not make it possible to control for confounders [23].

### Stage of primary disease

Twenty-six studies described the stage of primary tumor at diagnosis of CRC. In these studies between 8 and 64 % had stage 4 disease, most of the studies reporting more than 30 %. Generally stage 3 disease was the most common among BM patients in the included studies. In studies eligible for pooling of data, the weighted mean of patients having stage 3 was 46.6 % and stage 4 was 36.2 % (Table 3).

**Table 3:** Summary of findings in our review on incidence and characteristics of patients with brain metastases from colorectal cancer

	All studies		Studies with CRC patients eligible for data pooling				
	No. studies	Range	No. studies	No. CRC Patients	No. BM patients	Range	Weighted mean (95 % CI)
Incidence of BM	36	0.4–23 %	16	100,825	1588	0.6–2.7 %	1.55 % (1.48–1.63 %)
Characteristics of BM patients							
ᅟMedian age (years)	20	55.7–73	7	52,591	716	55.7–73	
ᅟMedian BMFI (months)	28	20–40	11	56,618	794	21–34.3	
ᅟMale	37	39–80 %	14	100,105	1578	44–80 %	57.2 % (54.7–59.8 %)
ᅟStage of primary disease	25		12	48,556	673		
ᅟ- Stage 1	21	0–11 %	11	43,211	531	0–11 %	1.3 % (0.5–2.8 %)
ᅟ- Stage 2	20	0–33 %	11	43,211	531	0–33 %	17.5 % (14.1–21.2 %)
ᅟ- Stage 3	22	17–77 %	11	43,211	531	32–77 %	46.6 % (41.9–51.2 %)
ᅟ- Stage 4	24	8–56 %	12	48,556	673	8–51 %	36.2 % (32.3–40.3 %)
ᅟRectal primary	31	14–71 %	13	57,288	799	20–67 %	48.5 % (44.9–52.0 %)
ᅟExtracranial metastases	31	5–100 %	11	55,840	779	77–100 %	87.7 % (85.1–90.1 %)
ᅟLung metastases	32	36–92 %	11	52,399	783	51–86 %	68.6 % (65.0–72.0 %)
ᅟLiver metastases	28	22–80 %	10	50,671	734	24–80 %	40.6 % (36.8–44.5 %)

*Abbreviations*: *CRC* Colorectal cancer, *BM* Brain metastases, *BMFI* brain metastases free interval (interval from primary diagnosis to BM development)

### Rectal location of primary tumor

Totally, 31 studies reported that the frequency of rectal cancer among patient with BM ranged from 14 to 71 %. Most studies reported a frequency of 40 to 60 %. Thirteen studies were eligible for pooling of data, and in these, 20 to 67 % of BM patients had rectal primaries, with a weighted mean of 48.5 %. Both autopsy studies reported that 41 % of BM patients had rectal primaries [9, 17].

A few studies tried to investigate whether rectal location was associated with BM. Hugen et al. reported a significantly higher incidence in rectal primaries (5 %) compared to colonic (2.6 %) among mCRC patients [9]. Sundemeyer et al. found a higher but not significantly increased incidence of BM in rectal cancer (4.4 vs 2.9 %) patients [25]. One study found that rectal location increased the hazard ratio, but not statistically significantly [36]. Interestingly, an old study by Chyun et al. in mCRC patients showed a higher incidence in CRC patients with right-sided tumor than left-sided. However, this study also reported a very high incidence of BM in the entire cohort (23 %), and their population might not reflect BM patients in general [27].

### Metastatic disease

Twenty-nine studies described the number of patients with extracranial metastases at diagnosis of BM. It ranged between 5 and 100 %. However, only one study reported 5 % [24], and all other reported a frequency higher than 63 %. Eleven of the studies eligible for pooling of data detailed how many of their BM patients had extracranial metastases, the incidence ranging from 73 to 100 %, with a weighted mean of 87.7 %.

A total of 32 studies described the prevalence of lung metastases at BM diagnosis and found it to range from 36 to 92 %, more than half of the studies reporting that 70 % or more patients had lung metastases. The 11 studies eligible for data pooling found that 51 to 86 % of BM patients had lung metastases at diagnosis, with a weighted mean of 68.6 %. In the autopsy study by Temple et al., 86.61 % of the patients had lung metastases at autopsy [17]. Three studies reported an incidence of BM in lung metastasis patients that ranged from 6.2 to 22.6 % [25, 37, 38]. A few authors investigated whether pulmonary metastases were associated with an increased incidence of BM by comparing patients with lung metastases and patients without lung metastases. Two studies showed that patients with lung metastases had a significantly increased risk of BM [25, 26]. Byrne et al. also reported an increased risk, but it was not significant [36], and Chyun et al. reported the prevalence of lung metastases to be 55 % in patients with BM compared to 27 % in patients without BM [27]. Hammoud et al. reported that lung metastases did not affect overall BMFI [19].

Twenty-eight studies reported a prevalence of liver metastases at BM diagnosis that ranged from 22 to 80 %, with half of the studies reporting less than 45 %. Ten studies were eligible for pooling of data. Here, 24 to 80 % had liver metastases at BM diagnosis, with a weighted mean of 40.6 %. The autopsy study by Temple et al. reported a prevalence of 76 % in BM patients at autopsy [17]. Six studies reported an incidence of 1.3 to 5 % after liver metastases. Four of these only included patients who previously had liver metastasectomy performed [33–36]. The two remaining reported an incidence of 2.5 and 2.9 % after liver metastases [25, 38]. Chiang et al. noted that the incidence of BM after liver metastases was significantly lower than after lung metastases [38]. Furthermore, Sundemeyer et al. noted a statistically significant decreased incidence of BM in patients with liver metastases compared to patients without liver metastases [25]. Chyun et al. reported a prevalence of liver metastases of 22 % in patients with BM compared to 80 % in patients without BM [27]. Liver metastases did not affect overall BMFI [19].

### Chemotherapy before BM development

Eleven studies included information about chemotherapy before BM were diagnosed. The number of patients who received chemotherapy before BM were diagnosed ranged from 53 to 92 % in the studies [12, 16, 19, 21, 24, 25, 43, 47, 51, 52, 59]. Sundemeyer et al. showed that the incidence of BM increased as the number of treatment lines increased, but this was not statistically significant [25]. Tanriverdi et al. did not find any association between amount of chemotherapy and incidence of BM [16].

### Biomarkers

*RAS* were the most investigated DNA mutations associated with BM. Mostly only *KRAS* was investigated, but two studies also included *NRAS* mutation analysis [26, 31]. Yeager et al. performed *RAS* mutation analysis in 918 CRC patients, and showed that patients with *NRAS* and/or *KRAS* mutations had statistically significant higher incidence of BM (6.1 vs. 1.9 % in wild type patients), even after controlling for age, tumor location and previous diagnosis of lung metastasis [26]. A study by Kemeny et al. in CRC patients who had hepatic metastases removed found the same association between *KRAS* mutation and BM, but the sample size was small and the association was not statistically significant [33]. Both studies found *KRAS* to be mutated more often in right-sided tumor than in left-sided [26, 33]. Tie et al. showed a significantly higher frequency of *KRAS*, but not *NRAS* mutation in BM patients compared to non-BM patients [31]. Additionally, two studies showed a higher prevalence of *KRAS* mutation than wild type in BM patients, but the sample sizes were too small for adequate statistical analysis [16, 48].

Ten studies analyzed carcinoembryonic antigen (CEA) in association with BM, and the majority found an increased level of CEA at BM diagnosis [7, 16, 18, 24, 36, 37, 40, 41, 50, 52]. Only Higashiyama et al. showed a potential predictive role of CEA. They reported a higher incidence of BM in patients with increased CEA level at pulmonary metastasectomy compared to patients with a normal level [37]. However, Byrne et al. did not find any association between CEA level increase and BM development [36]. Cancer antigen 19.9 (CA19.9) level was found to be elevated before BM development in a study by Tanriverdi et al., but no further analysis was made regarding this discovery [16].

Mutation in *PIK3CA* has also been proposed as a predictor of BM development. Yeager et al. found an increased incidence of BM in *PIK3CA* mutated patients compared to wild type, but most of the mutated BM patients also had *RAS* mutation, which made the interpretation difficult [26]. Tie et al. found an increased prevalence of *PIK3CA* mutation in BM and lung metastases compared to liver metastases, but could not show any significant association between *PIK3CA* mutation and BM development [31]. Two studies looked at *BRAF* as a potential predictor of BM. Tran et al. showed an increased incidence of BM in *BRAF* mutated compared to *BRAF* wild-type, but the association was not statistically significant [29], and Tie et al. did not find any association between BRAF mutation and BM [31]. Neural cell adhesion molecule (NCAM) has only been investigated in one small study, which showed significantly increased expression in primary tumors of BM patients compared to non-BM patients [64]. Epidermal growth factor receptor (EGFR) expression has also been investigated in one study, but only five BM patients were included, of whom two had EGFR expression in their BM [44]. Finally, C-X-C chemokine receptor type 4 (CXCR4) expression was investigated in a study by Mongan et al. CXCR4 expression in primary tumors was seen in 100 % of 11 BM patients, and only 50 % of ten patients without BM [11]. Maglio et al. presented a study in which they found O-6-methylguanine-DNA methyltransferase (MGMT) methylation to be elevated in 64.2 % of patients with BM, with high concordance with primary tumors and independent of *KRAS* mutation status. They compared this with results from older studies showing lower level of methylation in CRC patients without BM [59].

## Discussion

In this study we reviewed the current literature to describe the incidence of BM from CRC, and to identify possible characteristics associated with BM development. Table 3 summarizes our findings.

BM are a quite rare event in CRC patients. The incidence of BM in CRC patients was reported to be anything from 0.6 to 3.2 % in the studies included in this review, and the weighted mean was 1.55 % (95 % CI 1.48–1.63 %). The difference reflects the small sample sizes and statistical variation as well as regional differences, with Asian studies generally reporting a lower incidence than studies from Europe and North America. The true incidence of BM might be higher than what has been reported in clinical studies because some patients had no symptoms, others did not receive brain scans because of short life expectancy, and still others were alive after the end of the study period. Autopsy studies, however, report an incidence of 0.9 and 2.7 %, which is comparable to what is found in clinical studies [9, 17]. It has been suggested that the incidence of BM from CRC is increasing due to better diagnostic options and CRC patients living longer, but so far this has remained speculative [2]. Smedby et al. showed that the incidence of BM from all cancer types increased from 1987 to 2006 from 7/100,000 to 14/100,000 [46]. A study by Schouten et al. did not, however, find any increase in the incidence of BM [22]. In this review, we did not see an association between years of data collection and incidence of BM from CRC.

BM are a late stage phenomenon in CRC, and naturally more common in patients who already have metastatic disease, with an incidence ranging from 2.5to 23 % [9, 12, 23, 26–29]. In the studies included in this review, BMFI was between 20 and 40 months, and was shorter in patients with stage 4 disease compared to stages 1–3 [19, 43, 48], and higher in patients receiving more therapy between primary diagnosis and BM than in those receiving less [19]. This was also consistent with the observation that the BMFI increased from the 1980s to the 2000s, probably resulting from better treatment and earlier diagnosis of primary disease [52]. The shorter BMFI observed in patients receiving less therapy could be a result of a selection of patients who develop BM shortly after primary diagnosis.

Only a few studies have reported patient age at primary CRC diagnosis; nevertheless, it is possible to conclude that patients with BM are usually younger than the average patient with CRC. In the studies included in our review, the median age at BM diagnosis was 62 years or less in half of the studies, and only a few studies reported it to be higher than 65 years. This can be compared with the median age at primary diagnosis in CRC patients being 67 years for men and 71 years for women according to the US SEER register, and a similar result was found by Hugen et al. in a European population [9, 65]. The young age could be a consequence of the long BMFI, which makes it more likely for a young and healthy individual to develop BM than an older patient with several comorbidities, or it might reflect a more aggressive disease in younger patients.

One observation drawn from this review is that more men than women develop BM, but this difference has not been investigated very thoroughly. The explanation for this observation is likely that more male than female patients develop CRC. Around 55 % of CRC patients are male according to the GLOBOCAN 2012 [1]. This small difference could possibly be the result of statistical variation, but it cannot be ruled out that being male increases the risk of BM, and new studies need to investigate this.

In the studies included in this review, more than half of the studies reported that 48 % or more of patients had rectal primaries, and in studies eligible for data pooling, the weighted mean was 48.5 %. This was a much higher number than what should be expected, since the incidence of colon cancer was normally reported to be three times higher than the incidence of rectal cancer [9, 65], and therefore rectal cancer seems to be associated with an increased risk of BM. However, this was only investigated in a few studies and data were conflicting [9, 25, 27, 36]. Opposing this theory are two studies that looked at patients with colon cancer only and found an incidence of 2.5–4 %, which was higher than the incidence found in CRC patients in general [39, 40].

Lung metastases have often been hypothesized to increase the risk of BM development, and CRC patients with lung metastases have an incidence of BM between 6.2 and 22.6 %, which is considerably higher than the average incidence of BM even in mCRC patients [25, 37, 38]. A few authors also showed that patients with lung metastases had a statistically significant increased risk of BM [25, 26]. However, liver metastases did not seem to increase the risk of BM, and the incidence after liver metastases was reported to be 1.3–5 % [25, 33–36, 38]. Patients with liver metastases might even have a decreased risk of BM compared to patients with lung metastases [25, 38]. In our review, we found that about 70 % of BM patients had lung metastases at diagnosis and about 40 % had liver metastases. This deviated from the normal pattern of metastases from CRC. In mCRC, liver metastases are found in 70 % and pulmonary metastases only in 30 % [9]. This suggests that there is a relationship between lung metastases and BM, whereas it looks like there may be a reverse relationship between BM and liver metastases.

Several authors have presented theories to explain the different metastatic patterns in patients with BM. The most common hypothesis is that the pattern reflects the vascular anatomy; the cancer can spread to the brain through three principle routes: 1. through the portal vein to the liver, and from there to the lung and thereafter brain. 2. through the cava vein directly to the lung and thereafter the brain. 3. through the vertebral plexus directly to the brain [40, 48, 53]. The hypothesis explains why fewer patients with liver than lung metastases develop BM as well as the shorter BMFI after lung metastases compared to liver metastases. It also explains why rectal primaries increase the risk of BM, since the rectum drains more often through the cava vein than the colon.

A different hypothesis is that different molecular patterns of the cancer explain the pattern of metastases [11]. *RAS* mutations are the most thoroughly investigated, and *RAS* mutations have been associated with both increased incidence of BM and lung metastases [16, 26, 31, 33, 48]. However, this does not seem to explain why more BM patients have rectal primaries than average CRC patients, since *RAS* mutations are more often found in right-sided colon tumors than in rectal tumors [26, 33]. The association between BM and mutation in *PIK3CA* and *BRAF*, expression of NCAM, EGFR, and CXCR4, MGMT methylation and increase in the tumor marker CA19.9 have also been investigated, but only in one or two studies each and on small samples, so the possible predictive potential is hard to determine [11, 16, 26, 29, 31, 44, 59, 64]. The tumor marker CEA has been found elevated at BM diagnosis in several studies [7, 16, 18, 40, 41, 50], but only one study showed a possible predictive role of CEA, while one did not [36, 37]. CEA is usually used to monitor patients with CRC during therapy [66], and CEA is elevated (above 5 ng/ml) in approximately 70 % of patients with metastatic disease [67]. None of the studies have shown that an increase in CEA observed in patients with BM differs from that seen in patients with extracranial progression. Therefore CEA should not be considered as a specific marker of BM development, but as a general marker of tumor activity. However, one could argue that in the absence of visible extracranial tumor progression and increased CEA, brain involvement should be suspected. Besides these biomarkers investigated in clinical studies and presented here, several potential biomarkers have been found in animal and in vitro models elsewhere [68].

Increased awareness of specific characteristics can potentially increase the chance of early diagnosis of BM, which may lead to lower total number of BM and better performance status, ultimately increasing the potential number of treatment options [4]. From our review, it is clear that BM from CRC is a rare event, and it is not necessary to screen all patients. However, in patients with rectal primary, lung metastases, and/or *KRAS* mutation, increased awareness of BM is advisable.

This study had some limitations. First, we only had access to published material from the included studies. Second, in many of the included studies it was not possible to determine whether patients were consecutively included or not. This, in combination with all studies being retrospective, leads to a risk of publication bias. Many of the papers presented incidence from a population spanning long periods of time, limiting the conclusion on temporal variations. Most of the present studies contained few BM patients, and did not have sufficient strength or the right study design to clarify which factors increase the risk of BM. We recommend that physicians enter new large scale prospective clinical studies, preferably as international collaborations, to determine risk factors for brain involvement.

## Conclusions

The incidence of BM from CRC ranges from 0.6 to 3.2 %, and it did not seem to increase over time. BM are a late stage phenomenon and patients are usually younger than the average CRC patient. Rectal primary, lung metastases and *KRAS* mutation are associated with an increased risk of BM, and increased awareness of brain involvement in patients with these characteristics is necessary.

### Availability of supporting data

The datasets supporting the conclusions of this article are included within the article.

## Abbreviations

BM: brain metastases
BMFI: brain metastases free interval
CA19.9: cancer antigen 19.9
CEA: carcinoembryonic antigen
CRC: colorectal cancer
CXCR4: C-X-C chemokine receptor type 4
EGFR: epidermal growth factor receptor
mCRC: metastatic colorectal cancer
MGMT: O-6-methylguanine-DNA methyltransferase.
NCAM: neural cell adhesion molecule
SRS: stereotactic radiosurgery
WRBT: whole brain radiation therapy
